# Highly Efficient Photon Upconversion in Self-Assembled Light-Harvesting Molecular Systems

**DOI:** 10.1038/srep10882

**Published:** 2015-06-09

**Authors:** Taku Ogawa, Nobuhiro Yanai, Angelo Monguzzi, Nobuo Kimizuka

**Affiliations:** 1 Department of Chemistry and Biochemistry, Graduate School of Engineering, Center for Molecular Systems (CMS), Kyushu University, 744 Moto-oka, Nishi-ku, Fukuoka 819-0395, Japan; 2 PRESTO, JST, Honcho 4-1-8, Kawaguchi, Saitama 332-0012, Japan; 3 Dipartimento di Scienza dei Materiali, Università Milano Bicocca, via R. Cozzi 53, 20125, Milano, Italy

## Abstract

To meet the world’s demands on the development of sunlight-powered renewable energy production, triplet–triplet annihilation-based photon upconversion (TTA–UC) has raised great expectations. However, an ideal highly efficient, low-power, and in-air TTA–UC has not been achieved. Here, we report a novel self-assembly approach to achieve this, which enabled highly efficient TTA–UC even in the presence of oxygen. A newly developed lipophilic 9,10-diphenylanthracene-based emitter molecule functionalized with multiple hydrogen-bonding moieties spontaneously coassembled with a triplet sensitizer in organic media, showing efficient triplet sensitization and subsequent triplet energy migration among the preorganized chromophores. This supramolecular light-harvesting system shows a high UC quantum yield of 30% optimized at low excitation power in deaerated conditions. Significantly, the UC emission largely remains even in an air-saturated solution, and this approach is facilely applicable to organogel and solid-film systems.

Photon upconversion (UC), converting low-energy photons to higher-energy photons, is a key method for overcoming the efficiency limits of sunlight-powered devices, including photovoltaic cells and photochemical hydrogen production. Conventional methods, such as second harmonic generation and multistep excitation of lanthanides, require light intensities that are orders of magnitude higher than solar irradiance, and their low conversion efficiencies detract from their appeal^1^. However, a promising strategy developed in the last decade is workable with non-coherent, low-intensity light sources; namely, the sensitized triplet-triplet annihilation-based photon upconversion (TTA-UC) with multicomponent donor (D)-acceptor (A) systems (Fig. 1a)^2^,^3^,^4^,^5^,^6^,^7^,^8^,^9^,^10^,^11^,^12^,^13^,^14^,^15^,^16^. This mechanism starts with the generation of donor (sensitizer) triplets (^3^D*) by intersystem crossing (ISC) from the photogenerated singlet state (^1^D*) and succeeding D-to-A triplet-triplet energy transfer (TTET) to form optically dark, metastable acceptor triplets (^3^A*). The subsequent diffusion and collision of two excited acceptor triplets generate a higher energy excited singlet state (^1^A*) through TTA^17^, from which the upconverted fluorescence is emitted.

Although the researchers have demonstrated the proof-of-principle improvement of photovoltaic and photochemical devices by integrating TTA-UC materials^18^,^19^,^20^,^21^,^22^, the insufficient TTA-UC efficiency and strict deaeration process for avoiding oxygen quenching of triplet state make them far from real-world applications. To overcome this situation, there are three major challenges with TTA-UC. First, it is desired to develop UC systems that display quantum efficiencies exceeding the current record of 26%^12^ (the theoretical maximum is defined as 50% for two-to-one photon conversion process^14^). Second, the rational design of molecular systems that work under low excitation light intensities is required so that the lower-energy component of sunlight can be upconverted. Third, TTA-UC systems that operate under aerobic conditions need to be developed, which requires to suppress massive quenching of the triplet excited states by molecular oxygen. In conventional TTA-UC systems, the donor and acceptor molecules are dissolved in low-viscosity solvents, where the UC emission is largely quenched by dissolved oxygen^4^,^5^,^12^,^14^. To avoid this, donor and acceptor molecules have been dispersed in solid polymers^10^,^21^,^23^ or viscous liquid mixtures^11^,^19^. However, the consequent slow diffusion of dye molecules in these matrices inevitably lowered the TTET and TTA efficiencies, which necessitated the use of undesirably high excitation power.

Here, we report the first supramolecular triplet-energy-harvesting system that shows energy-migration-based TTA-UC instead of the conventional molecular-diffusion-based mechanism. In contrast to the extensive applications of singlet energy transfer and migration in supramolecular assemblies^24^,^25^, studies on triplet energy migration have been largely limited to the field of solid-state physics^26^,^27^,^28^ and photofunctional polymers^29^,^30^,^31^. Although energy-migration-based UC emission has been recently reported by us and others^13^,^16^,^32^,^33^,^34^,^35^,^36^,^37^, there exist no systems that fulfill all of the aforementioned requirements. To achieve these goals, it is imperative to improve the migration rate and range of triplet excitons and to develop molecular designs for reducing the collision with oxygen molecules. We have designed a novel amphiphilic acceptor **1** (Fig. 1b) that possesses a solvophobic multiple-amide-substituted 9,10-diphenylanthracene (DPA) unit and solvophilic ether-linked alkyl chains that are connected *via* L-glutamate linkers. DPA has been employed as a benchmark acceptor in TTA-UC^4^,^9^,^12^,^14^. The amide group-enriched L-glutamate connector has been employed to impart chirality and hydrogen bonding networks that improve control of molecular orientation and thermal stability^25^,^38^,^39^. The acceptor **1** self-assembles in organic media that efficiently uptake donor Pt(II) octaethylporphyrin (PtOEP) molecules, giving supramolecular donor-acceptor nanohybrids. They show efficient donor-to-acceptor TTET as well as fast triplet energy migration among closely-assembled acceptors, leading to a high UC quantum yield at low excitation power. Remarkably, the TTA-UC emission is mostly preserved even under air-saturated condition. Furthermore, the in-air TTA-UC emission was also observed in different important material forms such as gels and solid films, demonstrating the generality of the supramolecular triplet-harvesting strategy.

## Results and discussion

**Figure 2:**
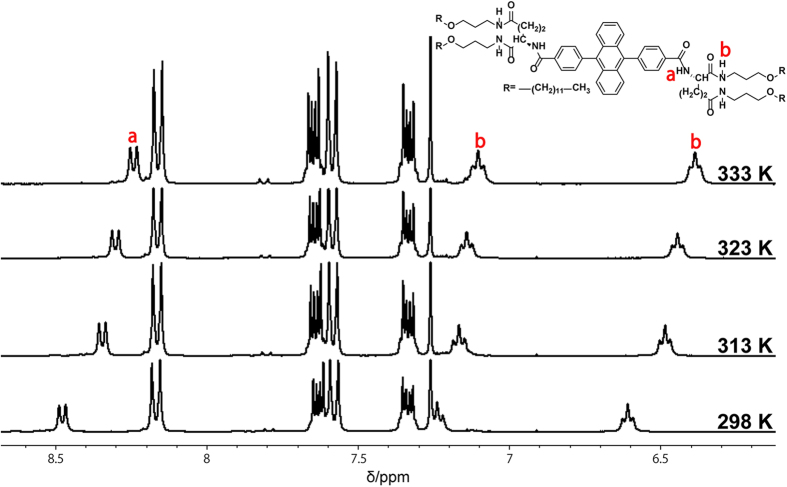
Supramolecular assembly of acceptor 1. ^1^H NMR spectra of **1** (10 mM in CDCl_3_, 300 MHz) at different temperatures. The protons attributable to the b position are split into two regimes because of their chiral environment.

The new acceptor **1** was synthesized and fully characterized, and its assembled structure in chloroform was studied by ^1^H NMR spectroscopy, absorption spectroscopy, and atomic force microscopy (AFM) measurements. ^1^H NMR spectra of **1** in deuterated chloroform (10 mM) showed amide proton signals at around 8.5, 7.2, and 6.6 ppm at 298 K, which shifted to a higher magnetic field upon heating to 333 K (Fig. 2). This result indicates the presence of hydrogen bonding interactions between the amide groups at 298 K, which were weakened by heating. Meanwhile, the ^1^H NMR peaks of the DPA unit did not show noticeable shifts upon changing the temperature, suggesting the absence of strong π-stacking interactions, which is ascribed to the steric hindrance imposed by the two out-of-plane phenyl rings. These considerations were further supported by the absorption spectra of **1** in chloroform (Supplementary Fig. 1), in which ^1^B_b_ and ^1^L_a_ absorption peaks of the anthracene chromophore did not show large changes upon heating. The AFM image of **1** drop cast of 1 mM solution on a highly oriented pyrolytic graphite (HOPG) surface exhibited tape-like nanostructures with an average width and height of around 20 and 2 nm, respectively (Supplementary Fig. 2). The latter value corresponds to the molecular length of a single acceptor molecule **1**, and thus **1** spontaneously self-assembles in chloroform to give developed monolayer membranes. Similar nanofiber structures were observed when an AFM specimen was prepared from a diluted solution ([**1**] = 20 μM), suggesting the structural stability even at lower concentrations (Supplementary Fig. 3). The fluorescence of **1** around 440 nm in chloroform gave a high absolute quantum yield of 88% ([1]  = 10 mM), which does not show a large overlap with the absorption band of PtOEP (Supplementary Fig. 4). The fluorescence quantum yield remained almost similar after diluting the solution for 1000 times (91%, [**1**] = 10 μM), which indicates the negligible concentration quenching of the acceptor singlet. This high quantum yield is obtained thanks to the structural characteristics of DPA. The steric hindrance of phenyl groups in the 9- and 10-positions prevents singlet quenching caused by strong intermolecular interactions and photo-dimerization^40^,^41^. Therefore, the use of DPA as the emitting unit is ideal to avoid the reduction of TTA-UC yield at high acceptor densities that has been observed for methacrylate copolymers containing 9-anthryl-methyl side-chains^42^.

**Figure 3:**
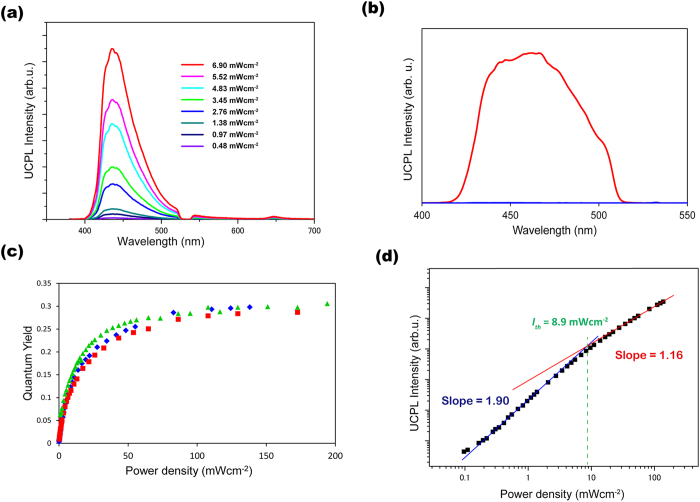
Highly efficient TTA-UC and low *I*_th_ value in the supramolecular system. (**a**) Photoluminescence spectra of **1** and PtOEP in deaerated chloroform ([**1**] = 10 mM, [PtOEP] = 10 μM) with different incident power densities of 532 nm laser at the room temperature. (**b**) Photoluminescence spectrum of **1**-PtOEP (red, [**1**] = 10 mM, [PtOEP] = 10 μM) and of DPA-PtOEP (blue, [DPA] = 10 mM, [PtOEP] = 10 μM) in air-saturated chloroform at 77 K under 532 nm excitation (excitation power density = 982 mW cm^−2^). (c) Dependence of UC quantum yield and on the incident power density in the deaerated condition at the room temperature ([**1**] = 10 mM, [PtOEP] = 10 μM). Different colors represent the data of different samples in the same condition. (d) Dependence of UC emission intensity at 440 nm on the incident power density in the deaerated condition at the room temperature ([**1**] = 10 mM, [PtOEP] = 10 μM).

The TTA-UC emission of a chloroform solution containing **1** and PtOEP ([**1**] = 10 mM, [PtOEP] = 10 μM) was measured after deaeration by repeated freeze-pump-thaw cycles. Under excitation at 532 nm, upconverted blue emission was clearly observed around 440 nm (Fig. 3a). The donor phosphorescence intensity observed at 650 nm is very weak, indicating that the TTET quantum efficiency *Φ*_*ET*_ is close to 1^43^. In general, the TTET process depends on molecular diffusion and collision, and thus the *Φ*_*ET*_ value decreases with the decrease in acceptor concentration^44^. However, in the case of the **1**-PtOEP pair, the *Φ*_*ET*_ value remained unchanged regardless of the degree of dilution (Supplementary Fig. 5). This behavior is explicable only by the unaltered donor-to-acceptor ratio in diluted supramolecular assemblies, clearly demonstrating their high structural integrity which arouse from the strong solvophobic binding of PtOEP molecules to the acceptor self-assemblies^16^. Importantly, the UC emission was clearly detected even at 77 K, well below the melting point of the solvent (Fig. 3b). A control experiment was carried out by using molecularly dispersed DPA instead of **1** under the identical conditions (same chromophore concentrations, excitation power, and optical setups). No upconverted emission was detected from the frozen chloroform solution of DPA-PtOEP at 77 K (Fig. 3b). At the given concentration, in the absence of molecular diffusion, energy migration and TTA are significantly limited, because of the large intermolecular distance between chromophores. These results confirm the presence of supramolecular structures, which allows triplet energy migration-based TTA-UC that is distinct from the classical molecular diffusion mechanism.

The UC quantum yield, *Φ*_*UC*_, was determined by using a tetrahydrofuran solution of DPA and PtOEP as the standard because they provide similar emission spectra to **1**, which allows to minimize the error induced by the optical response of the instrumental setup at different wavelengths (Fig. 3c)^12^. Because the TTA-UC process converts two photons to one photon with higher energy, the theoretical maximum of *Φ*_*UC*_is defined as 50%^14^. Significantly, the *Φ*_*UC*_ value of our system reached 30%, exceeding the previous highest value of 26% reported for the pair consisting of DPA and PtOEP ([DPA] = 10 mM, [PtOEP] = 100 μM)^12^. To confirm this result, we repeated the same measurements using different batches of **1** (Fig. 3c). The standard solutions were freshly made for each measurements. The quantum yield of 30 ± 1% with small error were repeatedly observed, which clearly ensures the high accuracy of our measurements and confirms the high quantum yield of 30%. The *Φ*_*UC*_ is described by the following expression^12^


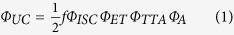


where *Φ*_*ISC*_, *Φ*_*ET*_, *Φ*_*TTA*_, and *Φ*_*A*_ represent the quantum efficiencies of donor ISC, TTET, TTA, and acceptor emission. The parameter *f* is the statistical probability for obtaining a singlet excited state after the annihilation of two triplet states. In the current system, the *Φ*_*ISC*_ and *Φ*_*ET*_ are regarded as 1, and *Φ*_*A*_ was experimentally determined as 0.88. The fluorescence lifetime of **1** in the presence of donor (5.3 ns) was identical to the one in the absence of donor (5.3 ns), indicating that the back energy transfer from acceptor to donor is almost negligible. At the saturation regime where *Φ*_*UC*_ is maximum and constant, the *Φ*_*TTA*_ can be assumed to be 1^21^. Thus the *f* value is calculated from Eq. 1 as 0.68, which is larger than the reported *f* value o*f* 0.52 for DPA^12^. We presume that the high *T*_2_ energy level and/or specific dye arrangement of the self-assembly system result in the observed enhanced *f* value. The TTA process is mediated by the formation of collisional complexes of singlet, triplet and quintet multiplicities, where the weighted statistical probability of formation of these states is 1 : 3 : 5^12^. Schmidt and coworkers reported that the pure statistical factor *f* = 1/9 can be enlarged to *f* = 2/5, because the quintet state of polyaromatic organic compounds is usually energetically inaccessible and the higher energy triplet product *T*_2_ decays back to *T*_1_^45^. This limit can be further broken when the *T*_2_ energy is even slightly higher than twice of the *T*_1_ energy^45^, as is the case for DPA (*f* = 0.52)^12^. The *f* value may also be affected by the mutual distance and orientation of adjacent acceptor moieties, since the interchange rate from *T*_1_-*T*_1_ dimer to *S*_1_-*S*_0_ state can be dependent on the intermolecular electronic coupling^46^.

Along with the maximum *Φ*_*UC*_, the TTA-UC threshold excitation intensity *I*_*th*_ is another important parameter characterizing the TTA-UC processes because it defines the excitation intensity at which *Φ*_*TTA*_ becomes 0.5^12^. The TTA-UC emission intensity generally shows quadratic and first-order dependences on the incident intensity in the low- and high-excitation intensity ranges, respectively, and the *I*_*th*_ value is experimentally determined as the intersection point of these two lines^45^,^47^,^48^. Such incident power dependence was observed for **1**-PtOEP (Fig. 3d), and it shows an *I*_*th*_ of 8.9 mW cm^−2^, which is close to the solar irradiance of 1.6 mW cm^–2^ at 532 ± 5 nm. To explain the observed low *I*_*th*_, we estimated the triplet exciton diffusion constant *D* in the supramolecular assemblies. The second order annihilation constant *γ*_*TT*_ of acceptor triplets was determined as 3.1 × 10^−11^ cm^3^s^−1^ by using the equation^47^


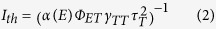


where *α(E)* is the system absorption coefficient in cm^−1^, and τ_*T*_ is the lifetime of the acceptor triplet measured using time-resolved photoluminescence experiments (Supplementary Fig. 6). Given that *γ*_*TT*_ = (8π*Da*_*0*_), by assigning *a*_*0*_ the value of 9.1 Å reported for the interaction distance of a DPA triplet pair^47^, we obtained a *D* value of 1.4 × 10^−5^ cm^2^ s^−1^. Significantly, this triplet energy migration rate in the acceptor assemblies is comparable to the large diffusion constant of DPA in a low-viscosity solvent (1.2 × 10^−5^ cm^2^ s^−1^)^44^ and to that observed for anthracene crystals^27^. This fast triplet energy migration in the self-assembly allowed the highly efficient TTA-UC process even under low excitation power. It is to note that the higher level of self-assembly achieved in this system reveals superior TTA-UC characteristics that surpass those observed for the amorphous, condensed liquid acceptor system (*I*_th_ ~ 50 mW cm^−2^)^13^.

**Figure 4:**
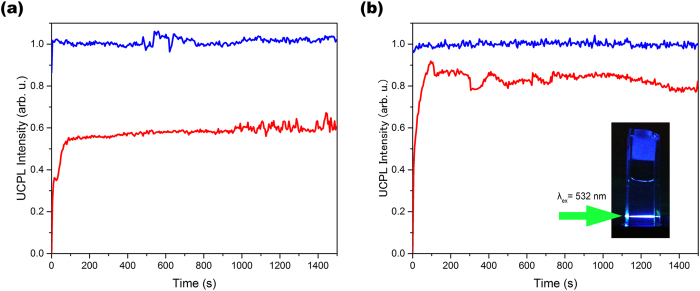
In-air UC emission of the supramolecular assembly. Time dependence of upconverted emission intensity at 435 nm upon continuous excitation at 532 nm of (**a**) DPA-PtOEP ([DPA] = 10 mM, [PtOEP] = 10 μM) and (**b**) **1**-PtOEP ([**1**] = 10 mM, [PtOEP] = 10 μM) in deaerated (blue line) and air-saturated (red line) chloroform at the room temperature. Excitation power density was 250 mW cm^−2^. Inset of (**b**): a photograph of the blue upconverted emission of the **1**-PtOEP pair in air-saturated chloroform.

We found that the UC emission of the current supramolecular system largely remained even in air-saturated dispersions (Supplementary Fig. 7). Before addressing its detail, we first explain results of a control experiment with DPA-PtOEP in chloroform. Prior to the preparation of air-saturated solutions, the solvent chloroform was bubbled with air for 1 h to make sure the complete saturation. Figure 4a shows time dependences of upconverted emission intensity under continuous excitation of DPA-PtOEP in deaerated and air-saturated chloroform. In the absence of oxygen, the UC emission intensity immediately reached to a certain value and remained almost constant. On the other hand, the UC emission intensity of the air-saturated solution showed a gradual increase for ca. 100 seconds, and then became constant. The observed gradual rise for the air-saturated solution suggests the consumption of oxygen molecules by chemical reactions^49^. The saturated UC emission intensity of the aerated solution showed a decrease of about 40% compared with the one of the deaerated condition, which is comparable to the recently reported behavior in DMF and DMSO^50^. Note that this quenching ratio would be dependent on solvents having different saturated oxygen concentrations. This observation implies the incomplete oxygen consumption in a cylindrical volume of the laser beam, and the physical/chemical quenching of the excited triplets by remaining oxygen^49^. Next, we compare these results of DPA-PtOEP with our supramolecular system. Similar to the case of DPA-PtOEP, the UC intensity of the deaerated **1**-PtOEP solution was constant from immediately after the light irradiation (Fig. 4b). In the air-saturated condition, again as is the case in the DPA-PtOEP pair, the time dependence of the **1**-PtOEP solution exhibited a gradual increase within 100 seconds, suggesting the consumption of surrounding oxygen molecules. To our interest, the **1**-PtOEP pair showed only an average of ca. 17% decrease by the presence of oxygen, which is much smaller than 40% quenching observed for DPA-PtOEP. This difference may be explained by lower concentration of remaining oxygen molecules in the local environment around the dye moieties in our supramolecular system as compared to the molecularly dispersed system, and this picture is consistent with the formation of closely-assembled array of chromophores. Further efforts to decrease local oxygen concentration around chromophore moieties based on the optimization of supramolecular architectures will provide a new opportunity to resolve the oxygen-related problems. Our current work offers a critical first step toward the realization of such ideal UC systems based on molecular self-assembly.

To achieve efficient donor-to-acceptor TTET in air-saturated systems, the location of donor molecules in the acceptor assembly is also crucial. To obtain this information, we measured the donor phosphorescence intensity and lifetime for frozen dispersions at 77 K (Supplementary Fig. 8). We confirmed that almost all of the donor molecules are bound to the acceptor nanoassemblies, where the donors show a bimodal distribution with shorter (4.8 Å, 35 mol%) and larger (17.3 Å, 65 mol%) average distances from the acceptor. At the ambient temperature, a fast equilibrium could naturally exist between these binding sites that allows efficient TTET to DPA units.

**Figure 5:**
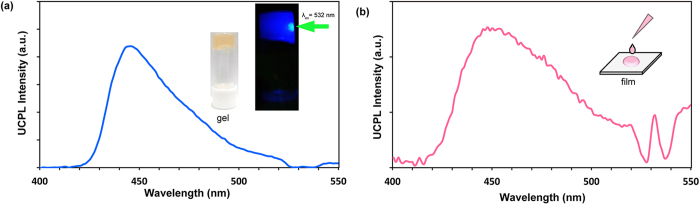
Generality of TTA-UC based on supramolecular chemistry. (**a**) Photoluminescence spectra of the 1,2-dichloroethane gel of **1** doped with PtOEP ([**1**] = 16 mM, [PtOEP] = 16 μM) upon 532 nm laser excitation in the ambient conditions. Inset pictures show the doped gel under white light and 532 nm green laser. (**b**) Photoluminescence spectra of the cast film of PtOEP-doped **1** (PtOEP/**1** = 0.1 mol%) obtained by 532 nm laser excitation in the ambient conditions.

The potential of the present supramolecular strategy can be extended beyond the dispersion systems. Supramolecular gels have recently received intense attention for designing photofunctional soft-materials including singlet energy harvesting systems^24^,^25^,^51^,^52^. The acceptor **1** was found to form organogels in 1,2-dichloroethane with a critical gelation concentration of 8 mM. Interestingly, the 1,2-dichloroethane gel of **1** doped with PtOEP showed a clear UC emission even in air, indicating that triplet excitons migrate in supramolecular gel networks to harvest the light energy and are upconverted in soft-materials (Fig. 5a). Furthermore, the self-assembly-based UC emission is also observed in solid cast films prepared by casting the chloroform solution under ambient conditions (Fig. 5b). This is surprising, since TTA-UC in crystalline system has long been suffered from the oxygen quenching issue and very limited miscibility of acceptor-donor pairs as well^12^. This is an important step towards the solid-state TTA-UC devices that operate even in air. These observations clearly confirm the value of the present self-assembly strategy, which would offer a general and powerful means to overcome longtime limitations in TTA-UC. The integration of self-assembly with TTA-UC thus pushes back the boundaries of the traditional methods and promises wide applicability in many disciplines.

## Conclusions

In this study, the highly efficient, low-power, in-air light-harvesting TTA-UC systems have been developed by introducing the concept of molecular self-assembly. The current work offers an important bridge between photon upconversion and supramolecular chemistry. The suitably designed amphiphilic acceptor molecules spontaneously self-assemble in organic media to give developed, nanotape-like monolayer assemblies. They effectively uptake donor molecules that leads to the highly efficient UC emission both in deaerated and aerated conditions. Various applications of the current light-harvesting system are conceivable, which would open new avenues to self-assembly-based molecular technology in many disciplines. The rational extension of light-harvesting self-assemblies to the other combination of chromophores will lead to novel near IR-to-visible or visible-to-UV upconversion systems for improving photovoltaic and photocatalytic devices. The design of amphiphilic light harvesting systems in water would find many biological applications. Beyond TTA-UC, we envisage the formation and migration of triplet excitons in various self-assemblies and their aerobic stability would exert great impacts on materials science.

## Methods

### Materials

All reagents and solvents were used as received otherwise noted. The detailed synthetic procedure and characterizations of the new acceptor **1** are written in the Supplementary Information. Platinum(II) octaethylporphyrin (PtOEP) was purchased from Sigma Aldrich. For spectroscopic measurements, we used analytical grade chloroform purchased from Wako Pure Chemical.

### Characterization

^1^H NMR (300 MHz) spectra were measured on Bruker DRX-300 spectrometer using TMS as the internal standard. Elemental analysis was conducted at the Elemental Analysis Center, Kyushu University. Atomic force microscopy (AFM, tapping mode) was carried out using an Agilent PicoPlus 5500. UV-vis absorption spectra were recorded on a JASCO V-670 spectrophotometer. Luminescence spectra were measured by using a PerkinElmer LS 55 fluorescence spectrometer. The samples were excited with an incidence angle of 45° to the quartz cell surface and the fluorescence was detected along the normal. The absolute quantum yields were calculated using a Hamamatsu C9920-02G instrument. Time-resolved photoluminescence lifetime measurements were carried out by using time-correlated single photon counting lifetime spectroscopy system, HAMAMATSU Quantaurus-Tau C11367-02 (for fluorescence lifetime)/C11567-01(for delayed luminescence lifetime). The quality of the fit has been judged by the fitting parameters such as χ^2^ (<1.2) as well as the visual inspection of the residuals. The upconversion luminescence emission spectra were recorded on Otsuka Electronics MCPD-7000 instrument with the excitation source using an external, adjustable 532 nm semiconductor laser.

### Determination of TTA-UC Emission Quantum Efficiency

The upconversion luminescence quantum efficiency (*Φ*_*UC*_) of a chloroform solution containing **1** and PtOEP ([**1**] = 10 mM, [PtOEP] = 10 μM) was determined relative to the DPA-PtOEP UC pair in deaerated tetrahydrofuran ([DPA] = 10 mM, [PtOEP] = 100 μM) according to the following equation^14^


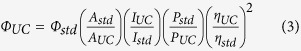


where *Φ*_*UC*_, *A*_*UC*_, *I*_*UC*,_*P*_*UC*_ and *η*_*UC*_ represent the quantum yield, absorbance at the excitation intensity (532 nm), integrated photoluminescence spectral profile, excitation power density, and refractive index of the medium. The corresponding terms for the subscript “*std*” are for the reference quantum counter of DPA-PtOEP pair in deaerated tetrahydrofuran at the identical excitation wavelength. The theoretical maximum UC quantum efficiency is 0.50 since the TTA-UC process uses 2 photons to produce 1 photon. The reflective index of chloroform and tetrahydrofuran are 1.44 and 1.41 at 293 K, respectively. The UC quantum yield of reference is known as 0.26 at its maximum^12^. Note that the comparison between different UC systems should be based on the careful examination of experimental conditions, since UC quantum yield of DPA-PtOEP is known to depend strongly on the experimental conditions, such as chromophore concentration, solvent, and excitation power, even if the employed donor and acceptor are identical^14^.

## Additional Information

**How to cite this article**: Ogawa, T. *et al*. Highly Efficient Photon Upconversion in Self-Assembled Light-Harvesting Molecular Systems. *Sci. Rep*. **5**, 10882; doi: 10.1038/srep10882 (2015).

## Supplementary Material

Supplementary Information

## Figures and Tables

**Figure 1:**
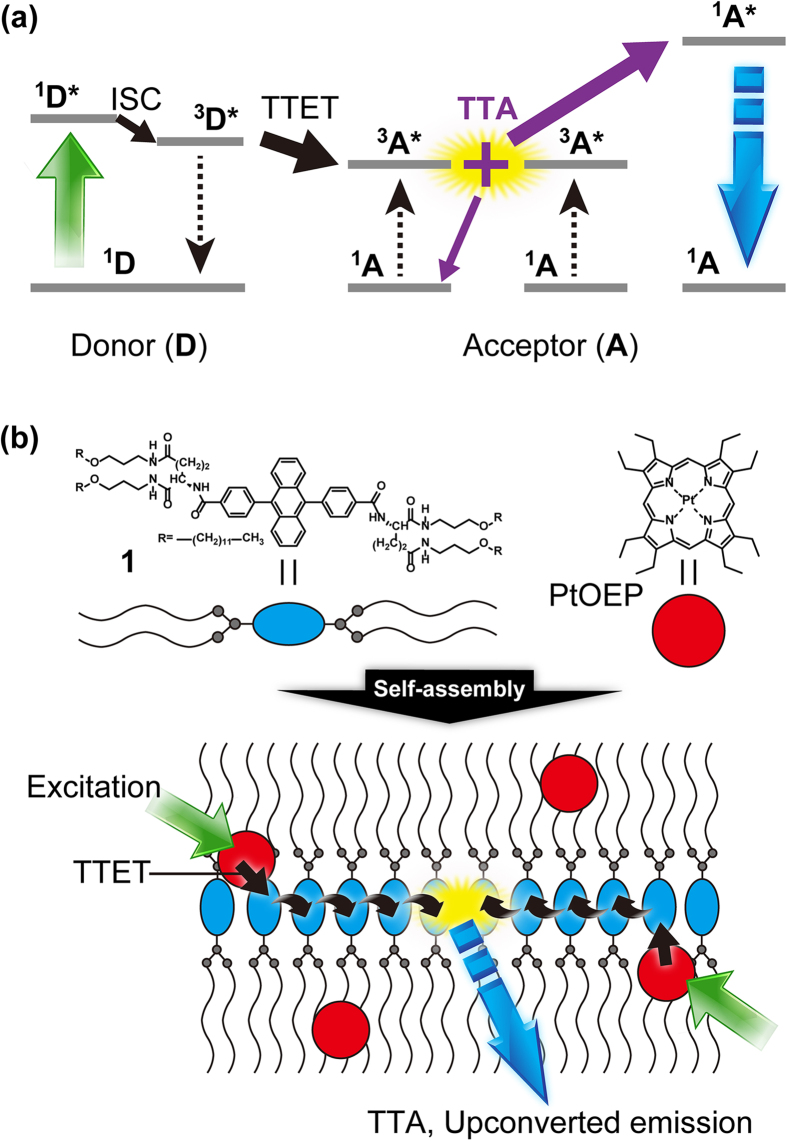
Schematic representations of the TTA-UC mechanism and the light-harvesting supramolecular TTA-UC system. (**a**) Scheme for the mechanism of TTA-UC. A triplet state of donor ^3^D*, formed by intersystem crossing (ISC) from the photo-excited (green arrow) singlet state ^1^D*, experiences triplet-triplet energy transfer (TTET) to an acceptor triplet ^3^A*. Two acceptor excited triplets annihilate to form a higher singlet energy level ^1^A*, which consequently produces upconverted delayed fluorescence (blue arrow). (**b**) Schematic illustration of the basic self-assembly structure. Acceptor molecules (**1**) spontaneously self-assemble in solution and donor molecules (PtOEP) efficiently bind to the self-assemblies by solvophobic interaction. Upon photoexcitation of donor molecules by green light, donor-to-acceptor TTET is followed by triplet energy migration among the acceptor networks. It leads to efficient TTA between acceptor triplets and consequent upconverted blue emission from the acceptor excited singlets.
