# Supplementary material for: Highly Efficient Photon Upconversion in Self-Assembled Light-Harvesting Molecular Systems
Source: Sci Rep. 2015 Jun 9;5:10882. doi: 10.1038/srep10882 (PMC4460878; doi:10.1038/srep10882)
Supplement: Supplementary Information [file srep10882-s1.pdf]

Supplementary information for

Highly Efficient Photon Upconversion in Self-Assembled Light-Harvesting Molecular Systems

Taku Ogawa,[1] Nobuhiro Yanai,*[1,2] Angelo Monguzzi,[3] and Nobuo Kimizuka*[1]

[1]Department of Chemistry and Biochemistry, Graduate School of Engineering, Center for Molecular Systems (CMS), Kyushu University, 744 Moto-oka, Nishi-ku, Fukuoka 819-0395, Japan

[2]PRESTO, JST, Honcho 4-1-8, Kawaguchi, Saitama 332-0012, Japan.

[3]Dipartimento di Scienza dei Materiali, Università Milano Bicocca, via R. Cozzi 53, 20125, Milano, Italy.

** To whom corresponding should be addressed. E-mail: yanai@mail.cstm.kyushu-u.ac.jp; n-kimi@mail.cstm.kyushu-u.ac.jp*

Synthesis of acceptor 1.

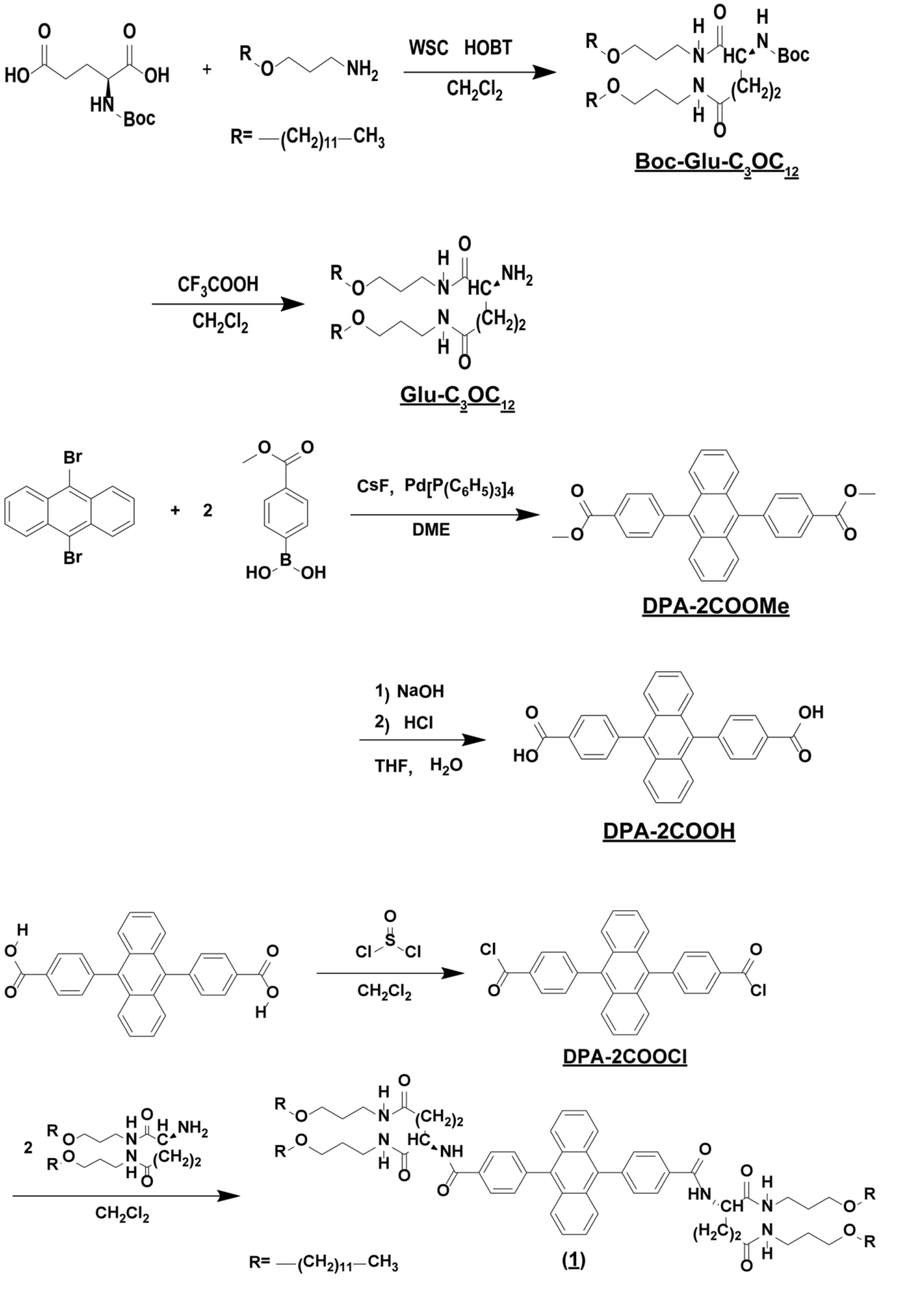

Synthesis of Boc-Glu-C$_3$OC$_{12}$. 3-(dodecyloxy)propylamine 12.7 ml (0.044 mol), 1-ethyl-3-(3-dimethylaminopropyl)-carbodiimide 8.43 g (0.044 mol), and 1-hydroxybenzotriazole 5.95 g (0.044 mol) were added to N-(tert-Butoxycarbonyl)-L-glutamic

acid 4.94 g (0.02 mol) in 400 ml distilled dichloromethane and reacted for 48h at the room temperature in Ar. After the reaction, the solution was washed several times with $NaHCO_3$ aqueous solution and with water, dried over anhydrous Na_2SO_4. Evaporation of the organic layer under reduced pressure followed by reprecipitation (methanol/water) and column chromatography (dichloromethane/methanol) over silica gel yielded the pure Boc-Glu-C_3OC_{12} solid (yield: 88%). ^{1}H NMR (300 MHz, $CDCl_3$): δ = 0.86-0.91 (t, 6H), 1.26 (m, 36H), 1.44 (s, 9H), 1.56-1.62 (m, 4H), 1.73-1.81 (m, 4H), 1.90-2.09 (m, 2H), 2.21-2.32 (m, 2H), 3.32-3.53 (m, 12H), 5.72-5.75 (d, 1H), 6.44 (t, 1H), 6.95 (t, 1H). ESI-MS: calculated for $C_{40}H_{79}N_3O_6$ 698.07; found 698.65 [M^+].

Synthesis of Glu-C_3OC_{12}. Boc-Glu-C_3OC_{12} 2.09 g (3.0 mmol) was dissolved in 150 ml of distilled dichloromethane and cooled by ice bath. Large excess of trifluoroacetic acid (6.88 ml) was added to this solution and stirred at the room temperature for 12 h. The solvent removal by reducing pressure gave an oily product. This product was dissolved in 10 mL THF and poured into 150 mL aqueous solution saturated with $NaHCO_3$. After filtration, the product was purified by recrystalization in methanol/ethyl acetate to give a colorless solid Glu-C_3OC_{12} (yield: 94%). ^{1}H NMR (300 MHz, $CDCl_3$): δ = 0.88-0.90 (t, 6H), 1.26 (m, 36H), 1.59 (m, 4H), 1.73-1.80 (m, 4H), 1.82-2.05 (m, 2H), 2.27-2.32 (m, 2H), 3.32-3.51 (m, 12H), 6.48 (t, 1H), 6.44 (t, 1H), 7.60 (t, 1H).

Synthesis of DPA-2COOMe.[1,2] A mixture of 2.12 g (11.8 mmol) 4-methoxycarbonylphenylboronic acid, 1.80 g (5.4 mmol) 9,10-dibromo anthracene, 2.60 g (24.2 mmol) CsF and 187 mg (0.61 mmol) Tetrakis(triphenylphosphine)palladium(0) were placed in a 300 ml flask under Ar, and 100 ml of degassed 1,2-dimethoxyethane were added. After refluxing under Ar for 60 h, the solvent was removed to give a yellow residue. This obtained solid was suspended in 35 ml water and extracted with 100 ml of $CHCl_3$. After drying the organic phase over Na_2SO_4 and removing the solvent, the product was purified by column chromatography ($CHCl_3$) over silica gel to yield of a yellow powder (yield: 21 %). ^{1}H NMR (300 MHz, $CDCl_3$): δ = 4.02 (s, 6H), 7.32-7.38 (m, 4H), 7.56-7.61 (d, 4H), 7.61-7.64 (m, 4H), 8.28-8.31 (d, 4H).

Synthesis of DPA-2COOH[1,2]. To a suspension of 288 mg (0.65 mmol) DPA-2COOMe in 75 ml 1:1 mixture of THF/MeOH, 15 ml of a 2M KOH aqueous solution was added. The mixture was allowed to reflux for 3 h. THF was removed under reduced pressure and the resulting suspension was diluted with water. The precipitate formed by acidification with aqueous HCl (2M) was collected by filtration, washed several times with water yielding 227 mg (84 %) of a pale yellow solid. ^{1}H NMR (300 MHz, d_6-DMSO): δ = 7.44-7.49 (m, 4H), 7.51-7.58 (d, 4H), 7.60-7.67 (m, 4H), 8.21-8.32 (d, 4H) 13.16 (s, 2H).

Synthesis of DPA-2(L)Glu (1). 3 ml of distilled benzene and 1 ml of thionyl chloride were added to 145 mg (0.35 mmol) of DPA-2COOH placed in a 10 ml flask under Ar. This mixture was refluxed for 3 h with catalyst quantity of DMF. After removing the solvent and excess thionyl chloride under reduced pressure, residual yellow solid was dispersed into 25 ml of distilled dichloromethane, and this solution was added dropwise to 623 mg (1.04 mmol) Glu-C_3OC_{12} dissolved in 25 ml of distilled dichloromethane under Ar. This mixture was stirred at the room temperature for 2h. After the reaction, the solution was washed several times with $NaHCO_3$ aqueous solution and with water, dried over anhydrous Na_2SO_4. Evaporation of the organic layer under reduced pressure and column chromatography (chloroform/methanol) over silica gel yielded the 403 mg (74%) of pale yellow solid. ^{1}H NMR (300 MHz, $CDCl_3$): δ = 0.81-0.92 (t, 12H), 1.16-1.38 (m, 74H), 1.49-1.57 (m, 8H), 1.73-1.90 (m, 8H), 2.14-2.46 (m, 2H), 2.50-2.68 (m, 2H), 3.34-3.47 (m, 16H), 3.47-3.58 (q, 4H), 4.59-4.71 (q, 2H) 6.45-6.52 (t, 2H), 7.17-7.25 (t, 2H), 7.30-7.40 (q, 4H), 7.55-7.61 (d, 4H), 7.61-7.67 (q, 4H), 8.13-8.20 (d, 4H) 8.44-8.51 (d, 2H). Elemental analysis: calculated for H 9.96 C 74.58 N 5.32; found H 9.93 C 74.40 N 5.25.

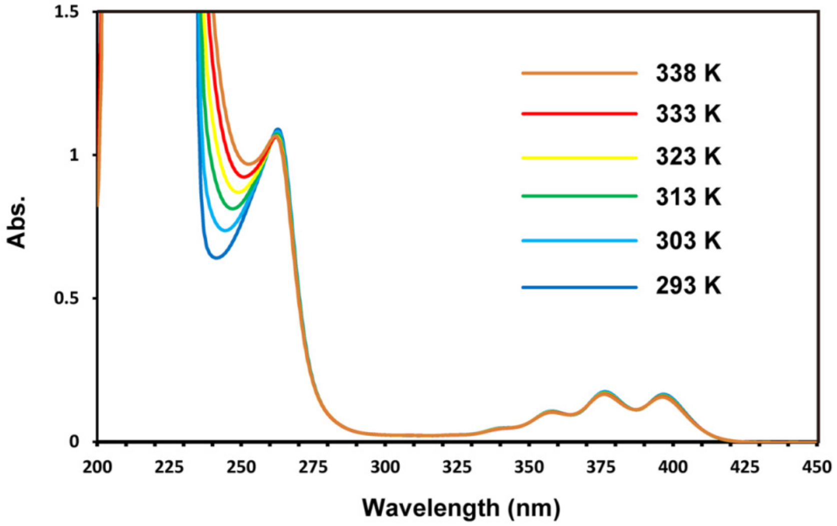

Supplementary Figure 1. Temperature-dependent absorption spectra of **1** in chloroform ([**1**] = 20 μM) between 293 K and 338 K. No large changes of the DPA absorption bands were observed with increasing the temperature. Similar results were observed by using 1 mM chloroform solution of **1**. These observations agree with the temperature dependent NMR results that suggest the absence of strong intermolecular interaction between the DPA moieties.

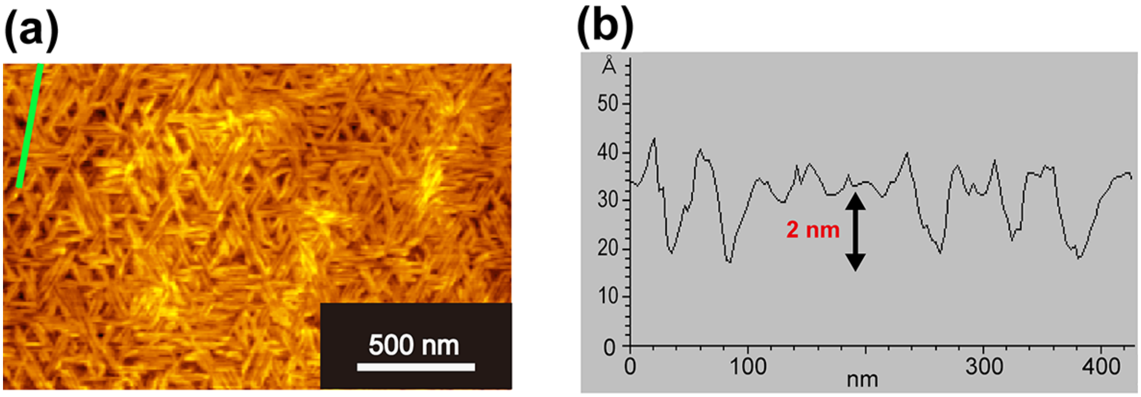

Supplementary Figure 2. (a) An AFM image of **1** drop-cast from 1 mM solution onto the HOPG substrate. (b) A height profile along the green line in (a).

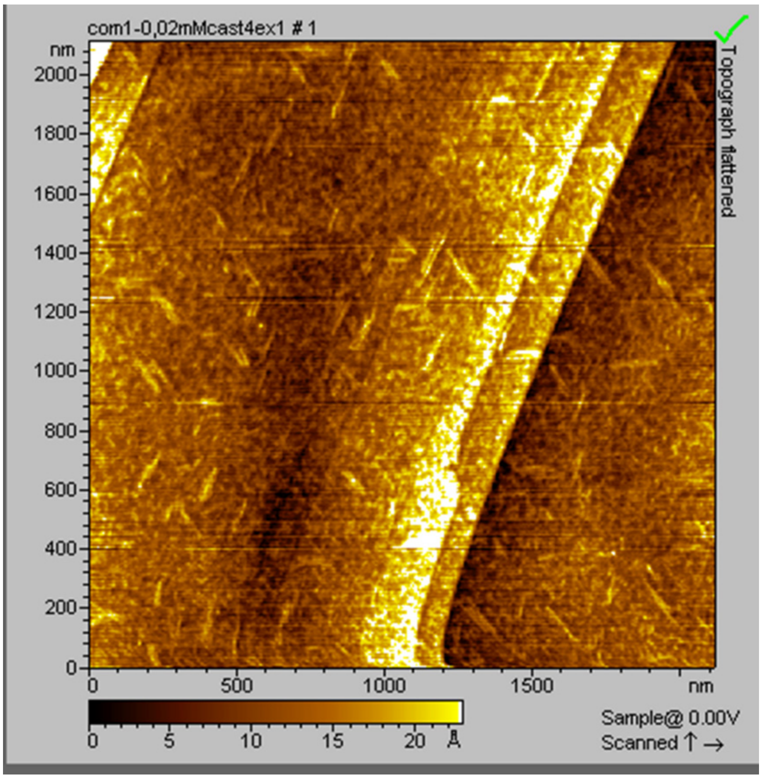

Supplementary Figure 3. An AFM image of **1** drop-cast from 20 μM solution onto the HOPG substrate.

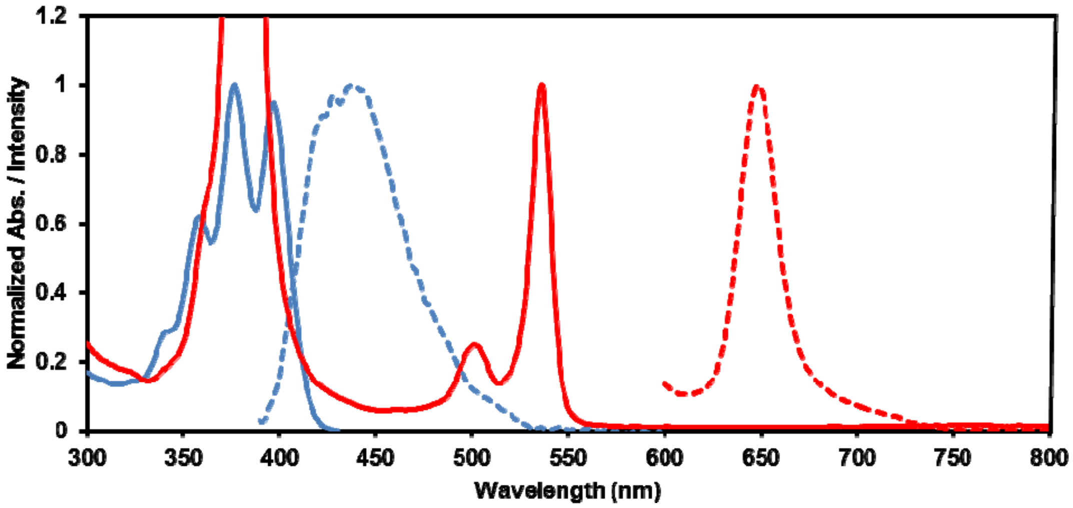

Supplementary Figure 4. Normalized absorption (solid lines) and emission (dashed lines) spectra of chloroform solution of **1** (blue, λ_{ex} = 375 nm, 1 mM) and PtOEP (red, λ_{ex} =510 nm, 10 μM) at the room temperature.

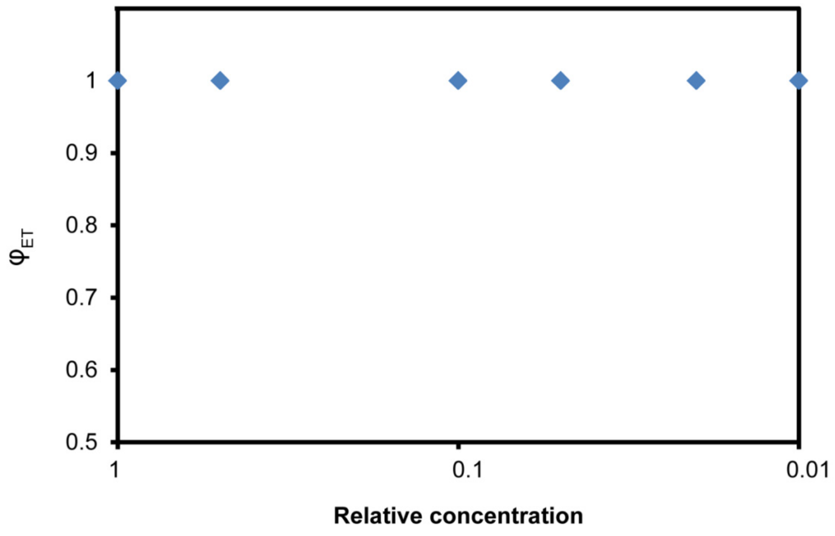

Supplementary Figure 5. The donor-to-acceptor TTET efficiency Φ_{ET} as a function of relative concentration of **1** and PtOEP at the room temperature. The initial concentrations were [**1**] = 10 mM and [PtOEP] = 10 μM, and the solution was diluted up to 100 times.

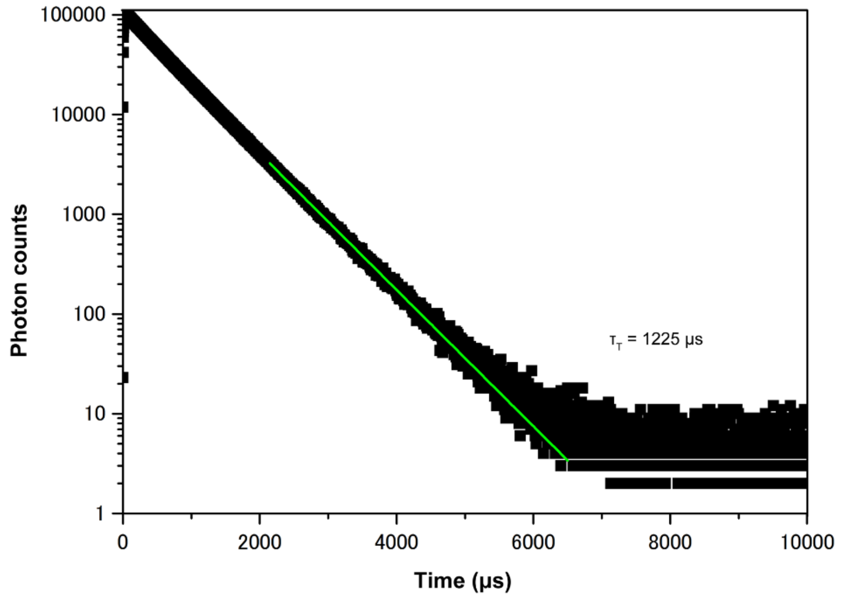

Supplementary Figure 6. The UC emission decays at 440 nm of **1**-PtOEP in deaerated chloroform ([**1**] = 10 mM, [PtOEP] = 10 μM) under 531 nm pulsed excitation at the room temperature. The fitting curves were obtained by considering the relationship of $I_{UC}(t) \propto \exp(-t/\tau_{UC}) = \exp(-2t/\tau_T)$, where τ_{UC} and τ_T are the lifetimes of the UC emission and acceptor triplet, respectively[3,4].

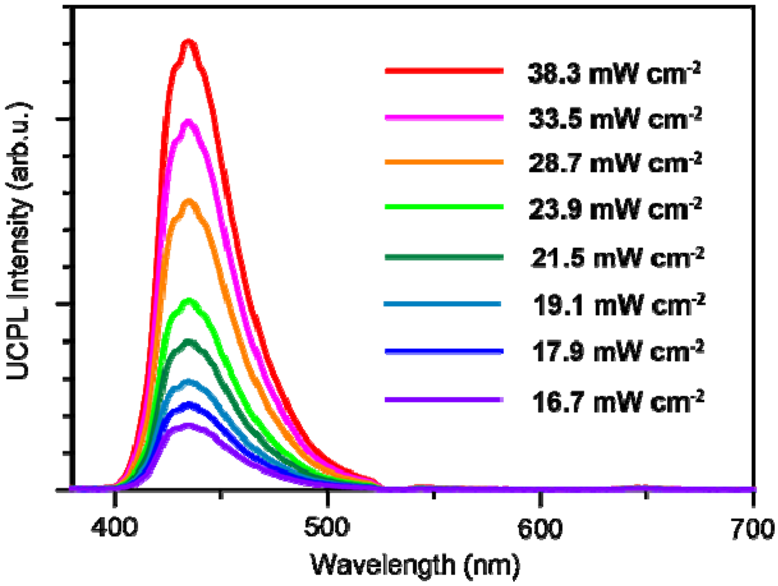

Supplementary Figure 7. Photoluminescence spectra of **1** and PtOEP in air-saturated chloroform ([**1**] = 10 mM, [PtOEP] = 10 μM) with different incident power densities of 532 nm laser at the room temperature. The shape of UC emission spectra in air is similar to those in the deaerated condition.

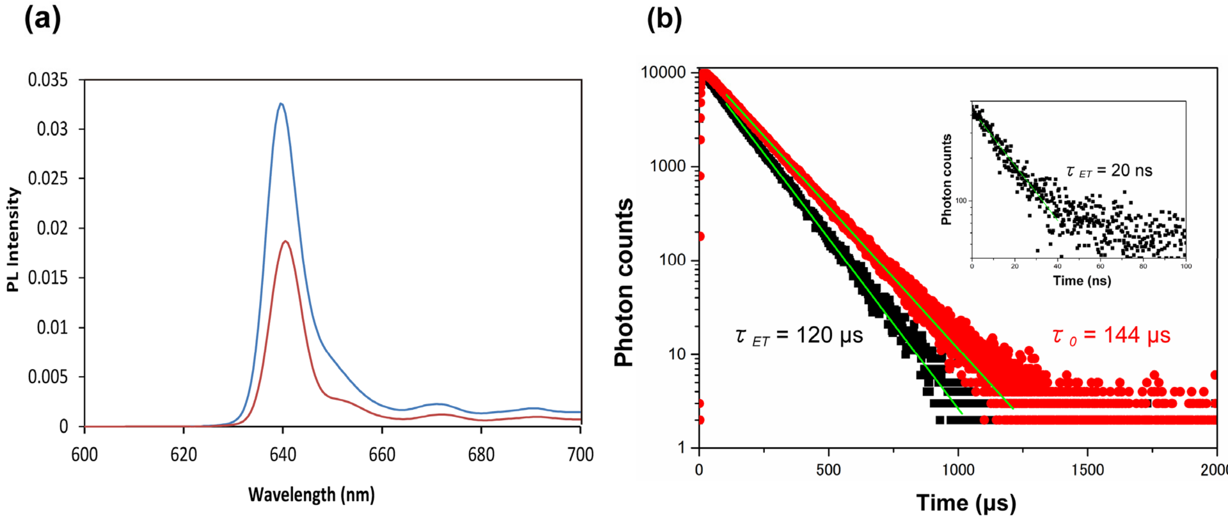

Supplementary Figure 8. (a) Phosphorescence spectrum of PtOEP with (red) and without (blue) acceptor **1** ([**1**] = 10 mM, [PtOEP] = 10 μM) in aerated chloroform at 77K under continuous excitation at 532 nm. (b) Phosphorescence decays of PtOEP with (black) and without (red) acceptor **1** ([**1**] = 10 mM, [PtOEP] = 10 μM; black) in aerated chloroform at 77 K under pulsed excitation at 531 nm. Inset: Nanosecond-scale phosphorescence decay at 650 nm of the **1**-PtOEP

chloroform solution ([**1**] = 10 mM, [PtOEP] = 10 μM) at 77 K with excitation of 365 nm.

The frozen PtOEP solution showed a mono-exponential decay with a lifetime τ_0 of 144 μs. In the presence of acceptor **1**, we observed a bi-exponential photoluminescence decay, with lifetimes τ_{ET} of 20 ns and 120 μs. No free PtOEP luminescence was detected, which means that almost all the donor molecules are accommodated in the acceptor nanoassemblies. By using the Perrin approximation for short range interactions[5], the donor-to-acceptor Dexter energy transfer efficiency can be written as a function of the center-to-center distance between donor and acceptor r_{D-A} by the equation

$$\Phi_{ET} = 1 - \exp\left[-\left(\frac{r_0}{r_{D-A}} \right)^3 \right] = 1 - \frac{\tau_{ET}}{\tau_0} \qquad \text{(S1)}$$

where r_0 is an effective interaction distance in the process energy transfer between PtOEP and DPA, being previously estimated as 9.8 Å[6]. The r_{D-A} values were calculated as 4.8 Å and 17.3 Å for the short (20 ns) and long (120 μs) τ_{ET} components, respectively. This suggests the spatial distribution of donor in the acceptor assemblies, taking into account the molecular length of the acceptor **1** is about 60 Å and it self-assembles into the nanotape-like structure of single molecular thickness. Moreover, it demonstrate that, by following Eq. S1, in a frozen systems where molecular diffusion is completely avoided, only the first-neighbors donor population (τ_{ET} = 20 ns) with (r_0/r_{D-A}) > 1 can efficiently transfer the energy to the acceptors (ϕ_{ET}= 1). For the second-neighbors donor population (τ_{ET} = 120 μs) with (r_0/r_{D-A}) < 1, the energy transfer yield is low (ϕ_{ET} = 0.17). The comparison of donor phosphorescence intensities between with and without acceptor **1** at 77 K provides the abundance ratio of 35 mol% and 65 mol% for the close and separated population, respectively.

Supplementary Reference

1 Ma, S. Q., Sun, D. F., Forster, P. M., Yuan, D. Q., Zhuang, W. J., Chen, Y. S., Parise, J. B. & Zhou, H. C. A Three-Dimensional Porous Metal-Organic Framework Constructed from Two-Dimensional Sheets via Interdigitation Exhibiting Dynamic Features. *Inorg. Chem.* **48**, 4616-4618 (2009).

2 Hauptvogel, I. M., Biedermann, R., Klein, N., Senkovska, I., Cadiau, A., Wallacher, D., Feyerherm, R. & Kaskel, S. Flexible and Hydrophobic Zn-Based Metal-Organic Framework. *Inorg. Chem.* **50**, 8367-8374 (2011).

3 Monguzzi, A., Bianchi, F., Bianchi, A., Mauri, M., Simonutti, R., Ruffo, R., Tubino, R. & Meinardi, F. High Efficiency Up-Converting Single Phase Elastomers for Photon Managing Applications. *Adv. Ener. Mater.* **3**, 680-686 (2013).

4 Pope, M. & Swenberg, C. E. *Electronic Processes in Organic Crystals*, (Oxford University Press, 1982).

5 Inokuti, M. & Hirayama, F. Influence of Energy Transfer by Exchange Mechanism on Donor Luminescence. *J. Chem. Phys.* **43**, 1978-1989 (1965).

6 Monguzzi, A., Tubino, R., Salamone, M. M. & Meinardi, F. Energy transfer enhancement by oxygen perturbation of spin-forbidden electronic transitions in aromatic systems. *Phys. Rev. B* **82**, 125113 (2010).
